# Supplementary material for: Genomic surveillance uncovers regional variation in HCV transmission networks in rural United States
Source: Nat Commun. 2025 Dec 2;17:249. doi: 10.1038/s41467-025-66934-y (PMC12783787; doi:10.1038/s41467-025-66934-y)
Supplement: Supplementary file 1 — Supplementary Information [file 41467_2025_66934_MOESM1_ESM.pdf]

Supplementary Information

Supplementary Table 1. Overview of samples sent to the GHOST laboratory for HCV sequencing.

**Sites**	**# Samples Sent**	**# QC Negative Samples ***	**# PCR failed**	**# Sequenced**	**# Passed GHOST QC Sequencing**
Illinois	13	6	6	1	1
Kentucky	437	97	58	282	251
New England	229	54	34	141	129
North Carolina	53	5	2	46	41
Ohio	106	30	8	68	64
Oregon	75	8	14	53	50
West Virginia	21	3	6	12	5
Wisconsin	267	90	9	168	151

* Samples denoted as QC negative are those below our limit of detection which means little if any viral RNA is present for amplification work.

Supplementary Table 2. Predictors of transmission cluster membership retained by LASSO penalized logistic regression. Variables were first selected using LASSO logistic regression with 10-fold cross-validation to optimize model performance. For interpretability, odds ratios (ORs), 95% confidence intervals (CIs), and P values were obtained by refitting an unpenalized logistic regression including only the LASSO-selected predictors. Reported *P* values are from two-sided unpenalized logistic regression refits. No adjustments for multiple comparisons were applied.

Variable	Odds Ratio	95% CI (lower)	95% CI (upper)	P value
Recruitment: Partner/spouse	0.94	0.21	4.13	0.934
Recruitment: Friend/associate	2.07	1.15	3.7	0.015
Recruitment: Person I use drugs with	1.49	0.82	2.72	0.19
Drug of choice: Methamphetamine (vs opioids)	0.6	0.37	0.99	0.046
Source of syringes: Drug dealer	0.35	0.12	1.06	0.063
Income source: Illegal	1.53	0.97	2.42	0.066
Age 18–29 (ref: 44–65)	1.53	0.96	2.44	0.074

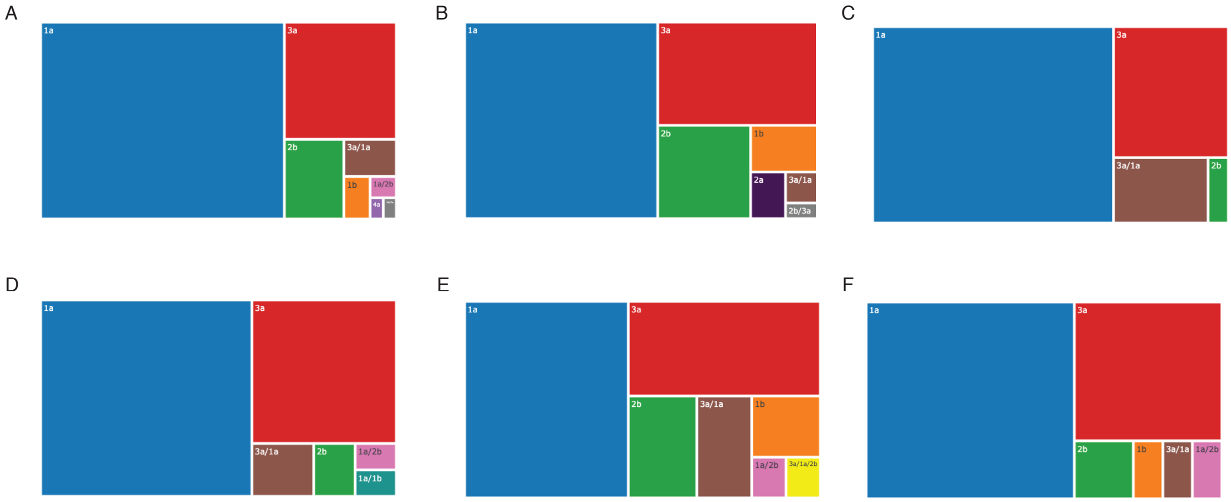

Supplementary Figure 1. Treemap overview of the diversity of HCV genotypes in study sites. The size of each genotype is proportional to the number of sequences detected in the lineage for **(A)** Kentucky, **(B)** New England, **(C)** Wisconsin, **(D)** Ohio, **(E)** Oregon and **(F)** North Carolina. Note West Virginia and Illinois are not shown due to the small number of sequenced samples.

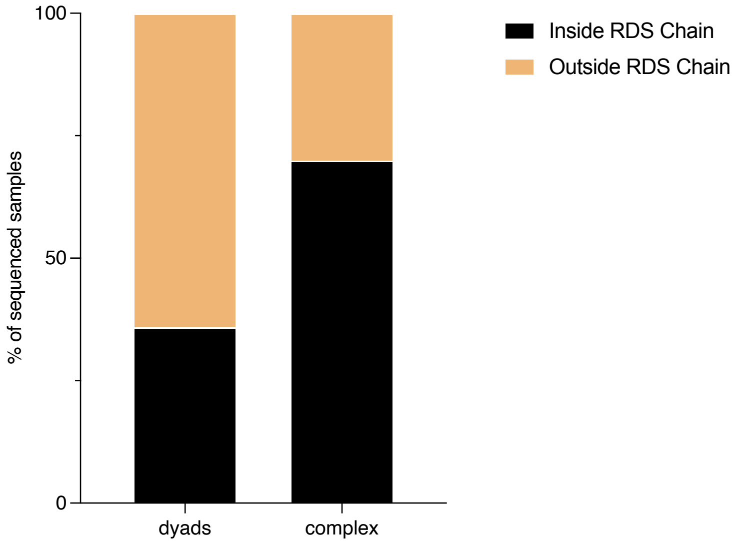

Supplementary Figure 2. Frequency of sequenced samples defined by cluster complexity and their relationship with RDS recruitment. Clusters are separated into dyads and complex networks.
