# Supplementary material for: Genomic surveillance uncovers regional variation in HCV transmission networks in rural United States
Source: Nat Commun. 2025 Dec 2;17:249. doi: 10.1038/s41467-025-66934-y (PMC12783787; doi:10.1038/s41467-025-66934-y)
Supplement: Supplementary file 3 — Reporting Summary [file 41467_2025_66934_MOESM3_ESM.pdf]

Corresponding author(s): Damien Tully
Last updated by author(s): Nov 6 2025

Reporting Summary

Nature Portfolio wishes to improve the reproducibility of the work that we publish. This form provides structure for consistency and transparency in reporting. For further information on Nature Portfolio policies, see our Editorial Policies and the Editorial Policy Checklist.

Statistics

For all statistical analyses, confirm that the following items are present in the figure legend, table legend, main text, or Methods section.

n/a	Confirmed	
☐	☒	The exact sample size (n) for each experimental group/condition, given as a discrete number and unit of measurement
☐	☒	A statement on whether measurements were taken from distinct samples or whether the same sample was measured repeatedly
☐	☒	The statistical test(s) used AND whether they are one- or two-sided<br>*Only common tests should be described solely by name; describe more complex techniques in the Methods section.*
☐	☒	A description of all covariates tested
☒	☐	A description of any assumptions or corrections, such as tests of normality and adjustment for multiple comparisons
☐	☒	A full description of the statistical parameters including central tendency (e.g. means) or other basic estimates (e.g. regression coefficient) AND variation (e.g. standard deviation) or associated estimates of uncertainty (e.g. confidence intervals)
☐	☒	For null hypothesis testing, the test statistic (e.g. F, t, r) with confidence intervals, effect sizes, degrees of freedom and P value noted<br>*Give P values as exact values whenever suitable.*
☒	☐	For Bayesian analysis, information on the choice of priors and Markov chain Monte Carlo settings
☒	☐	For hierarchical and complex designs, identification of the appropriate level for tests and full reporting of outcomes
☒	☐	Estimates of effect sizes (e.g. Cohen's d, Pearson's r), indicating how they were calculated

Our web collection on statistics for biologists contains articles on many of the points above.

Software and code

Policy information about availability of computer code

Data collection	Sequencing software included that from a MiSeq Illumina platform.
Data analysis	fastuniq v1.1, trimmomatic v0.36, V-FAT v 1.1, iVar 1.3, V-Phaser v 2, InStrain v 1.3.1, MAFFT v7.470 , IQ-TREE v 2.1, Augur v 24.3.0, BEAST v 1.10.4

For manuscripts utilizing custom algorithms or software that are central to the research but not yet described in published literature, software must be made available to editors and reviewers. We strongly encourage code deposition in a community repository (e.g. GitHub). See the Nature Portfolio guidelines for submitting code & software for further information.

Data

Policy information about availability of data

All manuscripts must include a data availability statement. This statement should provide the following information, where applicable:

- Accession codes, unique identifiers, or web links for publicly available datasets
- A description of any restrictions on data availability
- For clinical datasets or third party data, please ensure that the statement adheres to our policy

Consensus sequence data are available from GenBank under accession numbers (PX261275 - PX261966).

Research involving human participants, their data, or biological material

Policy information about studies with human participants or human data. See also policy information about sex, gender (identity/presentation), and sexual orientation and race, ethnicity and racism.

Reporting on sex and gender	Gender was considered as all participants were asked to identify their gender based on male, female, transgender, other or refused. Reporting on sex and gender was self-reported.
Reporting on race, ethnicity, or other socially relevant groupings	Race and ethnicity were considered as mutually exclusive categories. Partcipants were asked to self-report what race they are<br>from:<br>1 = White<br>2 = African American or Black<br>3 = American Indian<br>4 = Alaskan Native<br>5 = Asian, Pacific Islander, or Native Hawaiian<br>6 = African<br>7 = Mixed race<br>8 = Other
Population characteristics	Demographic characteristics were considered to establish the factors associated with being in a cluster.
Recruitment	Study participants were recruited between January 2018 and December 2021. Individuals were eligible for inclusion if they lived in the study area, reported any past 30-day injection drug use and/or noninjecting opioid use "to get high" (heroin, prescription pain medication). Inclusion criterion for all sites was age18 years except two states (Illinois, Wisconsin) where the age criterion was15 years. All sites conducted recruitment using respondent-driven sampling to facilitate sampling of hard-to-reach populations. Each study site identified "seed" participants to initiate recruitment chains. Seeds were recruited from syringe service programs, local health departments and community outreach to represent the general demographic characteristics of the local eligible population. Seeds recruited up to six members of their drug use network. Each referred participant recruited their network peers similarly with the goal of maximizing recruitment chains. Participants received $10 to $20 per successfully enrolled peer and $40 to $60 for completion of study procedures. Informed consent was obtained from all participants.
Ethics oversight	All participants provided written informed consent prior to sample or data collection at all participating institutions. All study procedures and protocols were approved by the Institutional Review Board at each participating site which were also reviewed and approved by the Institutional Review Board of Massachusetts General Hospital.

Note that full information on the approval of the study protocol must also be provided in the manuscript.

Field-specific reporting

Please select the one below that is the best fit for your research. If you are not sure, read the appropriate sections before making your selection.

☒ Life sciences ☐ Behavioural & social sciences ☐ Ecological, evolutionary & environmental sciences

For a reference copy of the document with all sections, see nature.com/documents/nr-reporting-summary-flat.pdf

Life sciences study design

All studies must disclose on these points even when the disclosure is negative.

Sample size	692
Data exclusions	A total of 1,201 HCV positive serum specimens were received from eight study sites. Of these 692 (57.7%) successfully completed sequencing and quality control while 293 (24.4%) samples were found to be below our limit of detection and contained little if any viral RNA. From 1,201 specimens, 216 (18.0%) either failed PCR or did not generate sufficient sequence to pass GHOST QC for inclusion. The PCR-only failure rate was 11.4% overall, varying by site (3.37–46.15%).
Replication	Not relevant
Randomization	Not relevant
Blinding	Not relevant

Reporting for specific materials, systems and methods

We require information from authors about some types of materials, experimental systems and methods used in many studies. Here, indicate whether each material, system or method listed is relevant to your study. If you are not sure if a list item applies to your research, read the appropriate section before selecting a response.

Materials & experimental systems

n/a	Involved in the study
☒	☐ Antibodies
☒	☐ Eukaryotic cell lines
☒	☐ Palaeontology and archaeology
☒	☐ Animals and other organisms
☒	☐ Clinical data
☒	☐ Dual use research of concern
☒	☐ Plants

Methods

n/a	Involved in the study
☒	☐ ChIP-seq
☒	☐ Flow cytometry
☒	☐ MRI-based neuroimaging

Plants

Seed stocks	*Report on the source of all seed stocks or other plant material used. If applicable, state the seed stock centre and catalogue number. If plant specimens were collected from the field, describe the collection location, date and sampling procedures.*
Novel plant genotypes	*Describe the methods by which all novel plant genotypes were produced. This includes those generated by transgenic approaches, gene editing, chemical/radiation-based mutagenesis and hybridization. For transgenic lines, describe the transformation method, the number of independent lines analyzed and the generation upon which experiments were performed. For gene-edited lines, describe the editor used, the endogenous sequence targeted for editing, the targeting guide RNA sequence (if applicable) and how the editor was applied.*
Authentication	*Describe any authentication procedures for each seed stock used or novel genotype generated. Describe any experiments used to assess the effect of a mutation and, where applicable, how potential secondary effects (e.g. second site T-DNA insertions, mosiacism, off-target gene editing) were examined.*
